# Superior Visible Photoelectric Response with Au/Cu_2_NiSnS_4_ Core–Shell Nanocrystals

**DOI:** 10.1021/acsami.3c17462

**Published:** 2024-02-26

**Authors:** Anima Ghosh, Shyam Narayan Singh Yadav, Ming-Hsiu Tsai, Abhishek Dubey, Chih-Ting Lin, Shangjr Gwo, Ta-Jen Yen

**Affiliations:** † Institute of Atomic and Molecular Sciences, Academia Sinica, Taipei 106, Taiwan R.O.C; ‡ Department of Physics, School of Sciences and Humanities, SR University, Warangal 506371, India; § Department of Materials Science and Engineering, National Tsing Hua University, No. 101 Section 2, Kuang Fu Road, Hsinchu City 300, Taiwan R.O.C; ∥ Graduate Institute of Electronics Engineering, National Taiwan University, Taipei 106, Taiwan, R.O.C.; ⊥ Department of Physics, National Tsing Hua University, Hsinchu City 300, Taiwan R.O.C; # Research Centre for Applied Science, Academia Sinica, Taipei 115, Taiwan R.O.C

**Keywords:** chalcogenides, core−shell nanocrystals, colloidal hot-injection methods, light-matter interactions, hybrid photodetectors

## Abstract

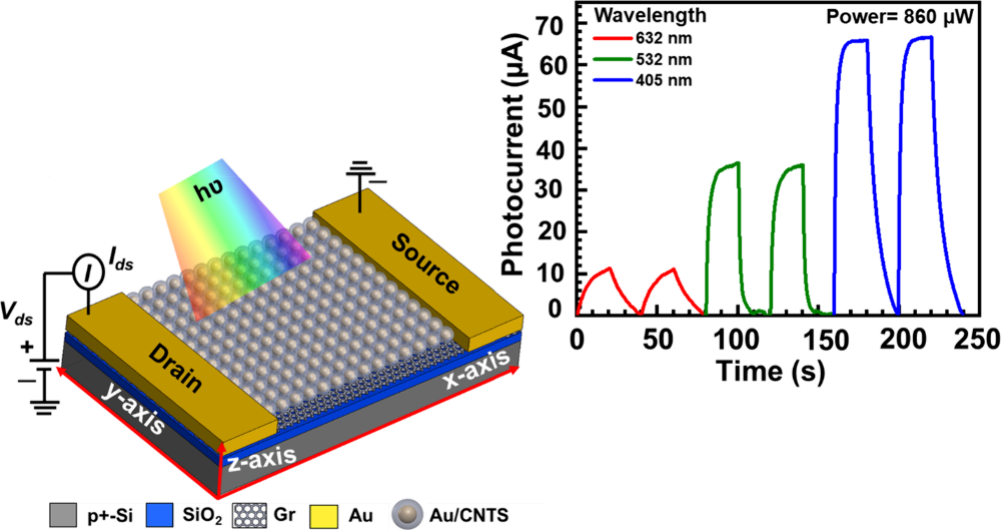

The incorporation of plasmonic metal nanostructures into semiconducting chalcogenides in the form of core–shell structures provides a promising approach to enhancing the performance of photodetectors. In this study, we combined Au nanoparticles with newly developed copper-based chalcogenides Cu_2_NiSnS_4_ (Au/CNTS) to achieve an ultrahigh optoelectronic response in the visible regime. The high-quality Au/CNTS core–shell nanocrystals (NCs) were synthesized by developing a unique colloidal hot-injection method, which allowed for excellent control over sizes, shapes, and elemental compositions. The as-synthesized Au/CNTS hybrid core–shell NCs exhibited enhanced optical absorption, carrier extraction efficiency, and improved photosensing performance owing to the plasmonic-induced resonance energy transfer effect of the Au core. This effect led to a significant increase in the carrier density of the Au/CNTS NCs, resulting in a measured responsivity of 1.2 × 10^3^ AW^–1^, a specific detectivity of 6.2 × 10^11^ Jones, and an external quantum efficiency of 3.8 × 10^5^ % at an incident power density of 318.5 μW cm^–2^. These results enlighten a new era in the development of plasmonic core–shell nanostructure-based visible photodetectors.

## Introduction

The development of earth-abundant chalcogenide materials has various applications such as spectroscopy, optoelectronics, and photovoltaics.^1−3^ Instead of conventionally expensive and toxic Cd- and Pb-based chalcogenide compounds, recent studies on copper-based chalcogenides, such as Cu_2_X(X = Zn, Fe, Co, Ni, Mn)SnS_4_ (i.e., CXTS), further draw extensive attention owing to their excellent optoelectronic properties, including p-type conductivity and direct band gaps of ∼1.2–1.5 eV.^4,5^ Moreover, a high absorption coefficient in the visible range^6−10^ makes them attractive as p-type photoabsorbing layers for optoelectronic devices.^11−18^ Among various CXTS compounds, Cu_2_NiSnS_4_ (CNTS) was found to exhibit an optical absorption coefficient of ∼10^6^ cm^–1^ and a very low conduction band offset (−0.12 eV).^4,5,7,19−21^ Furthermore, the optical properties of chalcogenide nanocrystals are significantly dependent on the chemical composition, crystal structure, particle size, and surface morphology, which could be controlled by synthesis methods. The proper amount of metal salt precursors together with chalcogen sources starts nucleation in a solution with a relatively low temperature, which makes their stabilization in nanocrystal (NC) form hugely amenable. Following the nucleation protocol, analogous low-dimensional Cu_2_NiSnS_4_ (CNTS) nanostructures were synthesized due to their large absorb surface and fast electron transport properties.^21^ In order to enhance optoelectronic responses, much attention has been paid in integrations of heterostructures, a combination of inorganic core–shell nanostructure,^22−24^ and the introduction of plasmonic resonances.^25^ So far, the practical applicability of CNTS suffers from its low optical absorption,^4,15^ which can be boosted by several methods already in use for other semiconducting materials. These methods include the hybrid of inorganic core–shell nanostructures^22−24^ and the introduction of plasmonic resonances.^25^ Especially, substantial efforts have been made to synthesize metal–semiconductor hybrid structures that offer unique interfacial electronic behaviors, superior band alignments, and excellent performance for optoelectronic devices.^16,19,21,26^ The metal/semiconductor hybrid nanostructures have opened up a new path by incorporating plasmonic metallic nanoparticles (NPs), which passivate surface states, thereby enhancing optical properties.^27−29^ Hence, these incorporations of plasmonic NPs in semiconductor NCs can improve charge extraction and collection,^30^ which is desired for achieving higher optoelectronic performance.^31−35^

In this work, we synthesized Au/CNTS core–shell NCs by developing a colloidal hot-injection method, which allows the tuning of the particle sizes and surface morphology to significantly enhance the optical properties.^36,37^ The Au core enhanced the optical absorption of the CNTS based on the plasmonic-induced resonance energy transfer (PIRET) effect, evidenced by the UV–visible absorption measurement and the finite difference time domain (FDTD) simulation. Such enhancement suggests that these photoactive core–shell NCs are excellent to use as photoabsorbent materials in the visible region for optoelectronic devices. Furthermore, we realized the first-ever Au/CNTS core–shell-based photodetector, demonstrating a high responsivity of 1.2 × 10^3^ AW^–1^, a specific detectivity of 6.2 × 10^11^ Jones, an external quantum efficiency (EQE) of 3.8 × 10^5^ % at an applied bias of 2 V, and a considerable response/recovery time of 3.4/13.0 s along with excellent operational reliability. The superior photoelectric responses are attributed to the outcome of the photoinduced photogating effects. Our results demonstrate the potential of plasmonic core–shell NCs for the development of visible photosensing devices for optoelectronic applications.

## Results and Discussion

**Figure 1:**
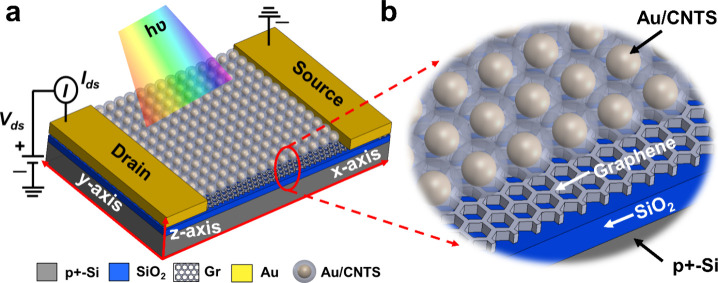
Schematic of the photodetector: (a) schematic illustration of Au/CNTS NCs and graphene-based hybrid photodetector. (b) Magnified view of the specifically chosen region of panel (a), showing core–shell Au/CNTS NCs on the top of monolayer graphene. Here, Au/CNTS works as a photoactive material, and graphene works as a highly conductive channel layer.

Our designed core–shell nanostructure and a graphene-based hybrid photodetector are illustrated in Figure 1a. Figure 1b presents a magnified view of the specifically chosen region from Figure 1a. In this hybrid photodetector, the core–shell nanostructure consists of the Au core as a plasmonic NP, enabling localized surface plasmon resonance (LSPR) and the shell CNTS nanostructure working as a photoactive semiconductor. Additionally, owing to high conductivity,^38^ ultrahigh charge carrier mobility (10^5^ cm^2^V^–1^s^–1^),^39^ and CMOS compatibility, graphene works as a channel layer to transport excited charge carriers under the applied bias voltage across the two electrodes. We synthesized CNTS and Au/CNTS NCs at a temperature of 200 °C using the colloidal hot-injection method^10,26^ in an inert atmosphere (refer to Experimental Methods for details). The optimized NCs were obtained by varying the growth time, thiol injection amount, controlling the temperature, and other related parameters. We observed that below the 200 °C temperature, the incomplete nucleation and growth process occurs due to which particles are in random shape and size, resulting in the impure phase of the NCs.

**Figure 2:**
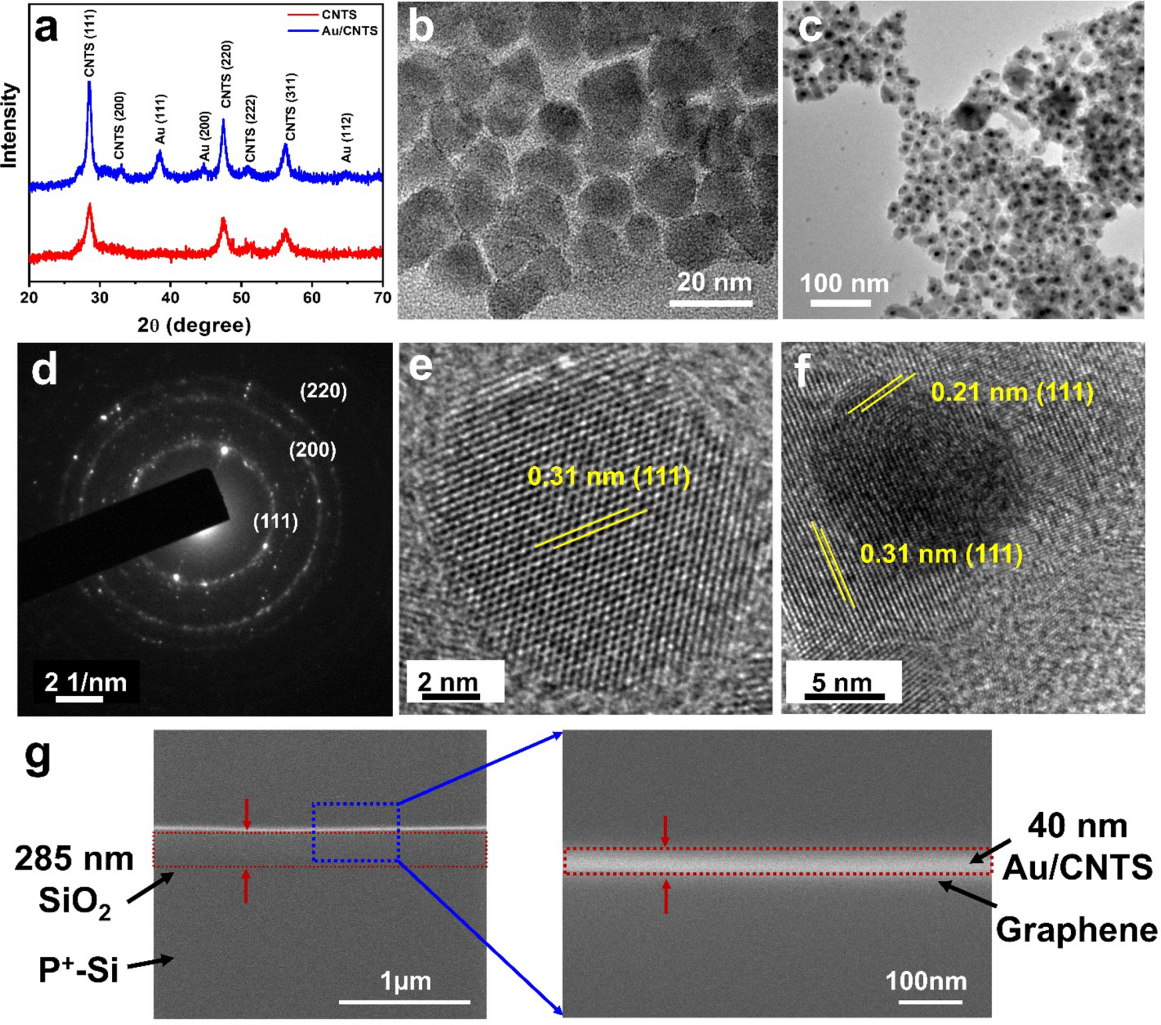
Structural characterization of CNTS, Au/CNTS NCs, and Au/CNTS-based device: (a) XRD patterns of CNTS and Au/CNTS NCs, indicating the pure cubic crystal structure of CNTS and face-centered cubic lattice of core Au. (b) TEM images of CNTS NCs indicating that the average particle size is ∼13.5 ± 2.4 nm. (c) TEM images of Au/CNTS NCs with 0.05 mmol of Au. (d) SAED pattern of a single CNTS NCs indicating that the NCs are polycrystalline and pure cubic crystal structure. The concentric rings correspond to the significant peaks observed in the XRD pattern. The scale bar shows the length scale in reciprocal space, i.e., nm^–1^. (e) High-resolution TEM image of CNTS NCs. (f) High-resolution TEM image of Au/CNTS NCs. The TEM image indicates that CNTS NCs and Au have interplanar distances of 0.31 and 0.21 nm, respectively. (g) FE-SEM image of a cross-sectional view of the fabricated Au/CNTS-based photodetector on a SiO_2_/Si substrate. The enlarged image is a cross section of Au/CNTS/SiO_2_.

Next, the structural and elemental compositions of these as-synthesized NCs were scrutinized using powder X-ray diffraction (XRD) spectroscopy, high-resolution transmission electron spectroscopy (HRTEM), energy-dispersive X-ray spectroscopy (EDXS), and X-ray photoelectron spectroscopy (XPS). The XRD spectra of CNTS and Au/CNTS NCs are shown in Figure 2a. The XRD spectral peaks of CNTS NCs at 28.43, 33.06, 47.49, 50.87, and 56.15° represent the (111), (200), (220), (222), and (311) planes, respectively. The XRD pattern confirms the pure cubic crystal structure of CNTS [JCPDS data no. 26-0552].^4,40^ Also, the XRD spectra (blue-colored line) for Au/CNTS core–shell NCs indicate the cubic phase of CNTS, and the cubic lattice of the Au core is evident from the (111) reflection of the face-centered cubic lattice. There are no prominent peaks caused by any impurities. The TEM images of CNTS and Au/CNTS NCs are exhibited in Figure 2b,c, respectively. Figure 2b reveals that the average particle size of CNTS NCs is ∼13.5 ± 2.4 nm. Figure 2c shows that the resulting NP of Au-incorporated CNTS is the core–shell structure of 0.05 M Au with CNTS, not the mixture or ad-mixture of individual Au nanoparticles and CNTS NCs.

The selected area electron diffraction (SAED) patterns are displayed in Figure 2d, indicating that CNTS NCs are polycrystalline and have a pure cubic crystal structure. The concentric rings of this SAED pattern agree well with the XRD analysis. The HRTEM images of CNTS NCs are shown in Figure 2e, demonstrating that the CNTS has an interplanar distance of 0.31 nm, which indicates the (111) plane of a cubic crystal. The HRTEM of Au/CNTS is shown in Figure 2f, demonstrating an interplanar distance of 0.21 nm corresponding to the (111) plane of the cubic Au NP in the core. The STEM image and elemental mapping of CNTS NCs are shown in Figures S1 and S2 (Supporting Information), displaying their distribution and validating the pristine CNTS and core/shell structure of Au NPs and CNTS NCs. The chemical compositions of CNTS and Au/CNTS NCs are verified from the EDXS analysis. The EDXS study shown in Figures S3 and S4 (Supporting Information) reveals that the atomic Cu/Ni/Sn/S ratio is close to the stoichiometry of Cu_2_NiSnS_4_. Furthermore, EDX analysis confirmed that the Au/CNTS core–shell NCs contained ∼5.24 wt % Au (Figure S4, Supporting Information) were prepared from 0.05 mmol of HAuCl_4_·3H_2_O. The cross-sectional views of the fabricated Au/CNTS-based device are shown in Figure 2g, demonstrating a clear Au/CNTS photoactive absorbing layer on the SiO_2_/Si substrate with a thickness of 40 nm.

The XPS measurements were performed on the surface of the NCs to determine the oxidation states of the constituent elements (Figure S5, Supporting Information). The XPS gives a suitable complement to EDX for chemical composition analysis. The Cu 2p core-level spectrum (Figure S5a, Supporting Information) shows peaks at 933.25 (2p_3/2_) and 953.15 eV (2p_1/2_) with a peak separation of 19.9 eV, confirming the Cu^+^ state. The Ni(II) peaks at 858.08 (2p_3/2_) and 875.24 eV (2p_1/2_) correspond to the Ni^2+^ state, with a splitting value of 17.16 eV (Figure S5b, Supporting Information). The doublet peaks of Sn(IV) are separate and located at 494.05 (3d_3/2_) and 485.05 eV (3d_5/2_), as shown in Figure S5c (Supporting Information). Figure S5d confirms the sulfur spectrum for the S^2–^ level from the 2p_3/2_ and 2p_1/2_ peaks with a doublet separation of ∼1.09 eV.

**Figure 3:**
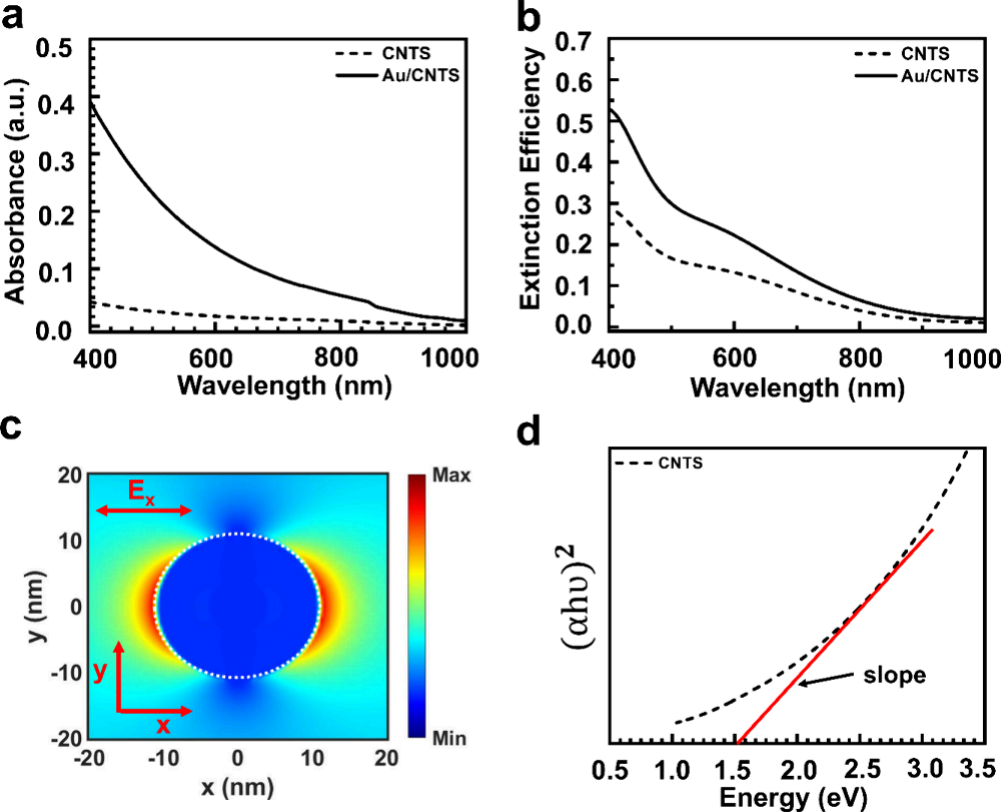
Optical characterization: (a) UV–visible absorption spectra for CNTS and Au/CNTS NCs. The optical absorption enhanced for Au/CNTS hybrid NCs in contrast with pristine CNTS NCs. (b) FDTD simulated extinction efficiency for CNTS and Au/CNTS NCs. (c) Electric field distribution *E*^2^ at a resonance wavelength of 405 nm for Au/CNTS NCs, indicating that an incident field localized around the NCs. (d) Tauc plot for band gap calculation of CNTS showing a bandgap of 1.54 eV.

Furthermore, we characterized the chemical vapor deposition (CVD)-grown graphene (for synthesis details, refer to Experimental Methods) using Raman spectroscopy, and the corresponding spectra are shown in Figure S6 (Supporting Information), demonstrating three feature peaks D, G, and 2D bands at 1340, 1580, and 2690 cm^–1^, respectively. The intensity ratio (*I*_2D_/*I*_G_) of the 2D and G peaks was found to be 2.34. Both the expected peak position and intensity ratio confirmed that the transferred graphene layer at the top of SiO_2_/p+-Si is a monolayer graphene.^41,42^ Next, we examined the absorption of both CNTS and Au/CNTS hybrid NCs to reveal the enhanced absorption efficiency by introducing Au NPs using the plasmonic effect. The corresponding UV–visible spectra are shown in Figure 3a. It is important to note that the pristine CNTS structure has a lower optical absorption efficiency in contrast to that of the Au/CNTS NCs. Both CNTS and Au/CNTS NCs exhibited a decrease in absorption efficiency with increasing wavelengths and an enhancement toward shorter wavelengths.

To study the enhancement in optical absorption due to the Au core in the CNTS shell, we performed a numerical simulation by using Lumerical software FDTD (refer to Experimental Methods for the simulation details). The scattering and absorption efficiency were calculated for both pristine CNTS and Au/CNTS hybrid NCs, as exhibited in Figure S7 (Supporting Information). The calculated extinction efficiency is depicted in Figure 3b and is in accordance with the measured UV–vis spectra for both CNTS and Au/CNTS NCs. The Au NP in the core dramatically enhances the absorption of the pristine CNTS NCs, in particular around its resonance wavelength because of the incident field localization caused by the induced LSPR. Additionally, there is an enhanced scattering efficiency due to the introduction of the Au core in the CNTS NCs, which increases the optical path length, resulting in high optical absorption.^28,43^ The electric field distribution was plotted at the highest absorption/extinction efficiency wavelength of 405 nm and is depicted in Figure 3c, illustrating that the electric field is highly confined around the NCs. This highly confined field induces hot electron generation, and the PIRET effect takes place from the surface of the metal to the semiconducting CNTS NCs simultaneously. Moreover, we draw a Tauc plot to calculate the band gap of synthesized pristine CNTS,^44^ depicted in Figure 3d. The band gap is calculated by intercepting the slope along the *x* axis and is found to be 1.54 eV, matching well with the reported literature.^4^

**Figure 4:**
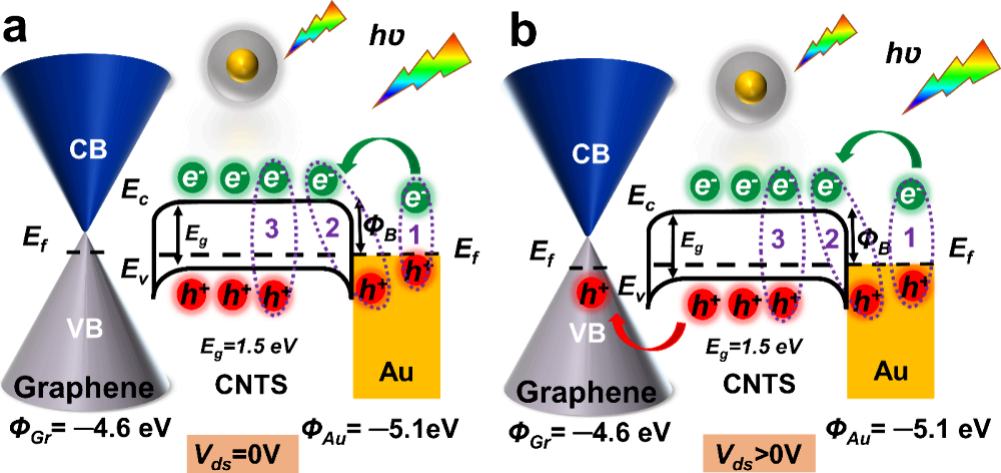
Energy band diagram of the Au/CNTS NCs/graphene. (a) Without applied bias *V*_*ds*_ = 0 V and (b) with applied bias (*V*_ds_ > 0 V). The valence band, conduction band, and Fermi level of CNTS NCs and p-type graphene are shown by *E*_v_, *E*_c_, and *E*_*f*_, respectively. The Φ_B_ indicates the Schottky barrier height. The symbols “h^+^” and “e^–^” denote holes and electrons, respectively. The purple-colored numbers (i.e., 1, 2, and 3) inside the dotted circle show the hot electron transfer, PICTT, and plasmonic-induced hot electron transfer effects, respectively. The green arrow indicates the hot electron transfer from the excited level of the Au NPs. The red arrow represents the charge carrier transfer from CNTS NCs to the graphene. Unlike the situation when *V*_ds_ > 0 V, there is no net current flow over the photodetector when *V*_ds_ = 0 V.

To explain the charge carrier generation and the transfer mechanism in core–shell Au/CNTS NCs and the graphene hybrid system, the schematics of the energy band diagram with an applied bias are illustrated in Figure 4. Here, the graphene has a Fermi energy of −4.6 eV,^45,46^ and Au has a work function of −5.1 eV. When a metal (Au) and a semiconductor (CNTS) are brought into contact, they form a Schottky barrier with a height of Φ_B_. At the Au NPs and CNTS interface, Au NPs plays an important role in enhancing the population of charge carriers in the CNTS NCs via nonradiative plasmonic decay as listed: (1) when light is incident on Au/CNTS NCs, plasmon-induced hot electrons are generated at the surface of Au and transfer to CNTS; (2) the plasmonic-induced charge transfer transition (PICTT) effect directly generates charge carriers in the conduction band of CNTS; and (3) the PIRET effect increased the population density of photogenerated charge carriers in CNTS NCs.^29^ These photogenerated charge carriers reach their corresponding energy level when *V*_ds_ = 0 V, and there will be no net current flow as depicted in Figure 4a. In contrast, under the applied bias across the electrodes, the charge carriers drift toward the opposite polarity and there will be a net current flow through the device, as shown in Figure 4b.

**Figure 5:**
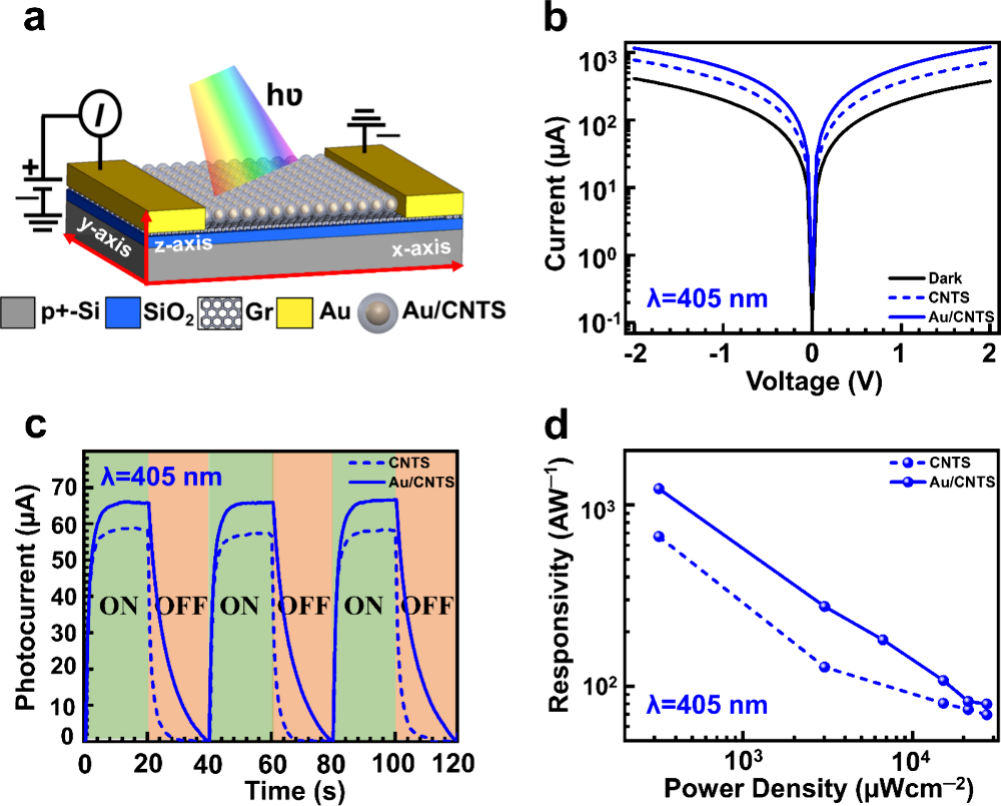
Optoelectronic characterization at 405 nm: (a) schematic of the Au/CNTS/graphene-based hybrid photodetector. (b) Current with applied voltage characteristic of CNTS and Au/CNTS-based hybrid photodetector at 860 μW incident power. (c) Transient photoresponse of CNTS and Au/CNTS-based photodetector at a constant incident power of 860 μW. (d) Responsivity for CNTS and Au/CNTS photodetector with respect to incident power density at a wavelength of 405 nm.

Next, we probed the synthesized Au/CNTS NCs optoelectronic characteristics and demonstrated its excellent performance as a visible photodetector. First, we fabricate two separate photodetectors using the bottom-up fabrication technique, as shown in Figure S9 (Supporting Information), using monolayer graphene as a conducting channel layer with pristine CNTS and Au/CNTS hybrid NCs as a photoabsorbing layer. The cross-sectional view of the designed photodetector is shown in Figure 5a. The photocurrent was probed using applied bias voltage (*V*_ds_) across two electrodes (drain and source).^47^ The dark/light current (*I*_dark_/*I*_light_) was measured without and with light illumination. The dark current is found to be of the order of μA due to thermally excited charge carriers at the surface of the graphene caused by the gapless band characteristic of graphene.^48^ The measured photocurrent (*I*_ph_ = *I*_light_ – *I*_dark_) and the transient response for the CNTS and Au/CNTS at an incident wavelength of 405 nm at a fixed illumination power of 860 μW are shown in Figure 5b,c, respectively. A significant photocurrent enhancement has been observed for the Au/CNTS core–shell NCs-based photodetector, in contrast to the pristine CNTS NCs-based photodetector.

The enhancement in the photocurrent can be understood by the following mechanisms: In plasmonic metal/semiconductor photodetectors, the intimate proximity between the metal and semiconductor facilitates efficient charge transfer owing to the pronounced localized surface plasmon resonance (LSPR) of the noble metal. This mechanism effectively mitigates the dipole–dipole coupling of excitons and plasmons, leading to reduced charge carrier recombination.^49^ These aforementioned mechanisms will help CNTS enhance the population of excited excitons. These excited excitons disassociated under an applied field across the two electrodes (drain and source) and drifted toward opposite polarities, resulting in the net photocurrents. To investigate the photoresponse characteristic of the photodetector, the key parameters, e.g., photoresponsivity (*R*_λ_), specific detectivity (D*), and the EQE, were calculated by the following (eqs 1–4):^50,51^
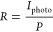
1
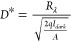
2
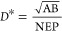
3
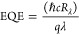
4

Here *I*_photo_, *P*, *R*_λ_, *q*, *I*_dark_, and *A* denote the photocurrent (*I*_photo_ = *I*_light_ – *I*_dark_), incident power, photoresponsivity at a wavelength of λ, electric charge, dark current, and active area of the device, respectively. Next, *B*, NEP, *h*, *c*, and λ denote the bandwidth, noise equivalent power, Planck constant, speed of light, and wavelength, respectively.
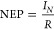
5

Here, *I*_N_ is the noise current and is defined as *I*^*2*^_N_ = *2qI*_dark_*B*.

The transient photocurrent response for both photodetectors was measured by applying a constant bias voltage *V*_ds_ of 2 V, as shown in Figure 5c. The results show that when the light was allowed to expose, the current increased with time, and it saturated after reaching its maximum due to the saturation in optical absorption and recombination of excess excitons that could not be collected through electrodes before they were annihilated. The calculated *R*_λ_ for pristine CNTS and Au/CNTS hybrid NC-based photodetector with the corresponding illumination power densities are shown in Figure 5d. Here, we observed that the responsivity for Au/CNTS hybrid NCs was enhanced in contrast to that of pristine CNTS NCs and decreased with higher illumination power density. The observed decrease in responsivity with increasing power can be attributed to optical absorption saturation, field screening by photoexcited carriers, and an elevated carrier scattering rate.^52−54^ To assess the photo response speed, we computed the rise time (τ_r_) and fall time (τ_f_). τ_r_ is characterized by the duration it takes for the maximum photocurrent to transition from 10 to 90%, whereas τ_f_ signifies the interval during which the maximum photocurrent decreases from 90 to 10%.^55^ The calculated rise/fall times for CNTS and Au/CNTS-based photodetectors were found to be 2.6/3.4 and 3.4/13.0 s, respectively, as shown in Figure S10. The rise and fall times mainly depend on the defect and trap densities of the bandgap region of semiconductors.^56,57^ The slow response time is mainly due to the photogating effect due to a trap state in the fabricated photodetector. The response time could be further improved by engineering the design of the photodetector.^58^

**Figure 6:**
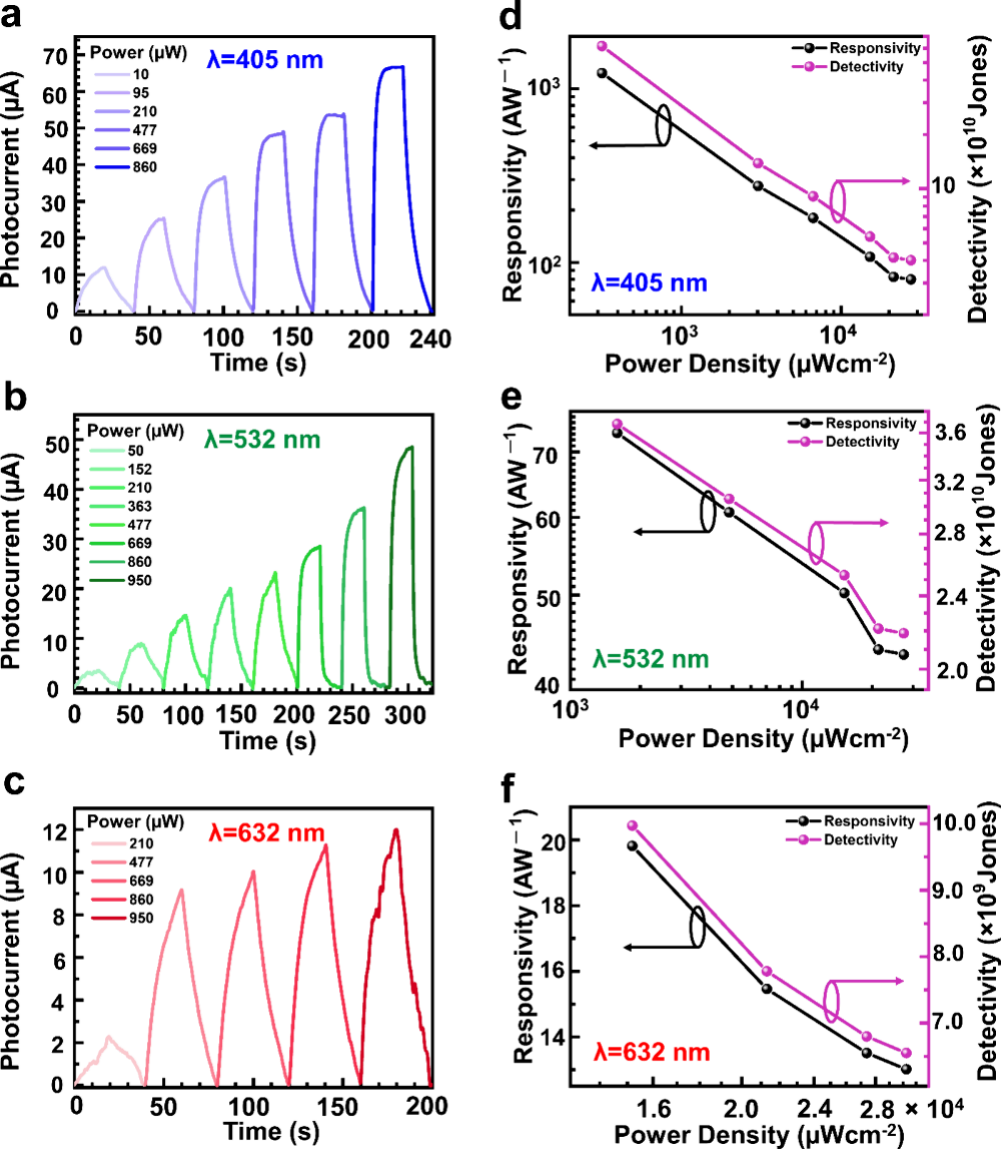
Optoelectronic characterization: (a–c) transient photoresponse for Au/CNTS-based hybrid photodetector with respect to the incident power at wavelengths of 405, 532, and 632 nm, respectively. (d–f) Photoresponsivity (*R*) in the black axis and specific detectivity (D*) in the pink axis for the Au/CNTS-based hybrid photodetector with incident power density at wavelengths of 405, 532, and 632 nm, respectively.

Furthermore, to explore the broadband response in the visible range of our fabricated photodetector, we conducted photocurrent measurements with three wavelengths of 405, 532, and 632 nm laser illumination under the visible spectrum. The time-dependent photocurrent with various incident power densities at a constantly applied voltage of 2 V is shown in Figure 6a–c. The calculated photoresponsivity and specific detectivity for 405, 532, and 632 nm wavelengths with illumination power density are exhibited in Figure 6d–f. The maximum photoresponsivity and detectivity were achieved at a 405 nm wavelength with the lowest incident illumination power because of the highest optical absorption at the shorter wavelength. The similar characteristics were observed for EQE (Figure S11, Supporting Information). It was found that the CNTS and Au/CNTS-based hybrid photodetectors are sensitive to all wavelengths in the visible spectrum.

**Figure 7:**
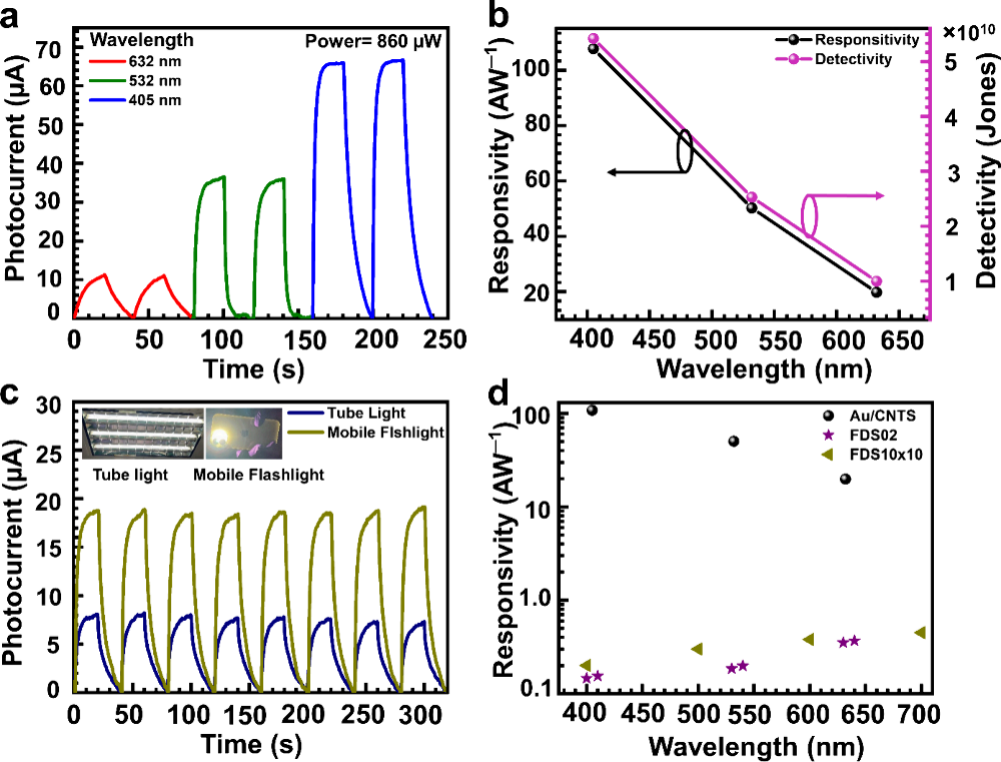
(a) Transient photocurrent response for the Au/CNTS-based hybrid photodetector with respect to time for incident wavelengths of 405, 532, and 632 nm at an incident power of 860 μW. (b) Wavelength-dependent photoresponsivity (*R*) (black axis) and specific detectivity (D*) (pink axis) for the Au/CNTS-based hybrid photodetector at a fixed incident power of 860 μW. (c) Multicycle transient photocurrent response of the Au/CNTS-based photodetector with incident light of daily used light sources (i.e., tube light and mobile flashlight). (d) Wavelength-dependent responsivity comparison plot of the Au/CNTS photodetector with commercial Si-based photodiode devices FDS02 and FDS10 × 10 (Thorlabs).

The photocurrent spectral response of the photodetector with a wavelength at a fixed illumination power of 860 μW is shown in Figure 7a. The corresponding calculated responsivity and detectivity with wavelength at a fixed illumination power are shown in Figure 7b. Our designed photodetector has a broadband response for visible light with the best performance at a 405 nm wavelength. The better optoelectronic performance (responsivity, detectivity, and EQE) at shorter wavelengths can be understood by UV–vis absorption spectra and extinction efficiency exhibited in Figure 3a,b, respectively. The results indicate that maximum photoelectric responses are achieved using an excitation laser of 405 nm. The superior photoelectric responses are achieved by the photoinduced photogating effect in the designed Au/CNTS/graphene photodetector.^58^ Next, to demonstrate the real-world application of the Au/CNTS-based photodetector, the multicycle transient photocurrent response under white light illumination using different light sources (i.e., tube light and mobile flash light) was measured and is shown in Figure 7c. The multicycle transient response with white light demonstrates that our fabricated device has potential to be used for real life application. Finally, the achieved optoelectronic characteristics were compared with commercial devices (FDS02 and FDS10 × 10 Si-based photodiode) and other reported works with a similar device configuration are shown in Figure 7c and Table S1 (Supporting Information), confirming that our Au/CNTS/graphene-based photodetector achieved superior optoelectronic performance among the Si-based photodiode and other copper chalcogenide-based photodetectors in the visible region.

## Conclusions

To summarize, we synthesized noble CNTS and Au/CNTS hybrid NCs. The XRD and HRTEM results show that our synthesized CNTS and Au possess a pure cubic and face-centered cubic crystal structure with interplanar distances of 0.31 and 0.21 nm, respectively. The XPS results indicate that synthesized nanocrystals constitute all of the elements with reasonable oxidation states. Furthermore, the optical studies of CNTS and Au/CNTS NCs indicate that Au/CNTS core–shell hybrid NCs possess significantly higher optical absorption than pristine CNTS NCs. Using these NCs, we fabricated a superior hybrid broadband photodetector and investigated its optoelectronic performance. The Au/CNTS/graphene-based photodetector achieved an ultrahigh responsivity of 1.22 × 10^3^ AW^–1^, specific directivity ( 6.18× 10^11^ Jones), and a large EQE of 3.76 × 10^5^ % at an incident wavelength of 405 nm. Notably, enhanced optoelectronic performance was achieved at a shorter wavelength because of enhanced optical absorption induced by the LSPR effect of the Au core in Au/CNTS NCs. The induced LSPR confines the incident field in the vicinity of the Au core and increases the population of excited charge carriers significantly, resulting in an enhanced photocurrent. Furthermore, contact optimization, interfacial engineering, and optimizing the photodetector’s design could achieve a better optoelectronic performance. This Au/CNTS core–shell NC-based photodetector has many potential optoelectronic applications.

## Experimental Methods

### Materials

Copper(II) acetylacetonate (Cu(acac)_2_, ≥ 99.9%), nickel(II) acetylacetonate hydrate (Ni(acac)_2_, 99.99%), tin(II) chloride (≥99.99% trace metals basis), gold(III) chloride (≥99.99%), *n*-dodecanethiol (*n*-DDT, ≥ 98%), *tert*-dodecanethiol (*t*-DDT, 98.5%), and oleylamine (OAm, ≥ 98%) were purchased from Sigma-Aldrich. In addition, hexane (fraction from petroleum), acetone, and ethanol (anhydrous, Merck) were used without further purification.

### Synthesis of Cu_2_NiSnS_4_ (CNTS) NCs and Au-Incorporated Cu_2_NiSnS_4_ (Au/CNTS) Core–Shell NCs

The colloidal hot-injection method was used to synthesize quaternary chalcogenide CNTS NCs and the Au/CNTS core–shell. In a typical synthesis of CNTS, 1 mmol of Cu(acac)_2_, 0.5 mmol of Ni(acac)_2_, and 0.5 mmol of SnCl_4_·5H_2_O were mixed with 10 mL of OAm in a three-necked flask and stirred under vacuum for 60 min at room temperature. Then, the system was backfilled with nitrogen, and the solution was subsequently heated up to 130 °C. The reaction was followed by a quick injection of a mixture of 1-DDT and *t*-DDT (1:1 ratio) under a nitrogen atmosphere with continuous stirring. Subsequently, the solution was heated up to 200 °C and maintained for the optimum time for completing the reaction. After the reaction, the mixture was cooled down to room temperature, and the final products were obtained via centrifugation with a mixture of hexane and ethanol. The as-synthesized NCs were readily dispersible in nonpolar solvents, e.g., octane, hexane, etc.

A similar procedure was adopted for the synthesis of Au/CNTS NCs using a Au NP solution. Then, 0.05 mmol of HAuCl_4_·3H_2_O was dissolved separately in oleylamine and heated to 120 °C with a continuous stirrer for 10 M for Au NP growth. Finally, Au NP solutions were rapidly injected into the Cu–Ni–Sn precursor in the flask. For photodetection device fabrication, the as-synthesized CNTS and Au/CNTS core–shell NCs were dispersed in *n*-hexane with a 50 mg/mL concentration (optimized).

### Graphene Synthesis and Transfer

CVD was utilized to synthesize a high-quality monolayer graphene with a large area on copper foil (99.8%, 25 μm thick) in a tabular quartz furnace. During the growth of monolayer graphene, the tube temperature was raised to 1000 °C in 80 min under a continuous 110 sccm H_2_ gas flow. While the tube was at 1000 °C, CH_4_ gas of 11 sccm flowed into the quartz tube and continued for 60 min for graphene to grow. The monolayer graphene can be procured on the Cu substrate after the furnace is cooled under ambient H_2_ gas and CH_4_ gas. Then, the monolayer graphene was transferred from Cu foil to a 300 nm SiO_2_/Si substrate using the method of electrochemical delimitation.^47^ A monolayer of graphene, grown using CVD, was transferred onto a precleaned SiO_2_/Si substrate through a wet transfer procedure involving acetone, isopropyl alcohol, and deionized water. Details are illustrated in Figure S8 (step II). The as-synthesized transferred graphene at the SiO_2_/Si substrate was characterized by Raman spectroscopy.

### Materials Characterization

XRD spectra were recorded with a Rigaku X-ray diffractometer (Cu Kα irradiation, λ = 1.541 Å). TEM, HRTEM, and HAADF-STEM images were taken from JEOL, JEM-2100F. For TEM analysis, the NCs were dispersed in hexane and were drop cast on 300-mesh Ni grids. For the FE-SEM cross-sectional view, samples were prepared using a focused ion beam of Ga source to cut the sample and glass needles were used to pick the sample and placed on TEM Cu grid. FE-SEM Hitachi SU8010 was used to capture cross-sectional images. UV–vis absorption spectra were recorded by using a Jasco V-670 spectrophotometer. The core-level XPS spectra of the NCs were obtained using Al Kα radiation (1486.6 eV) with a base pressure of 1.2 × 10^–8^ Torr in the photoelectron spectrometer PHI 5000 Versa Probe II, FEI Inc. and was calibrated against C 1s core-level spectra at 284.8 eV at a base pressure of 5 × 10^–11^ Torr. Raman spectra of the CVD-grown graphene were measured by using micro-Raman spectroscopy (HORIBA, LabRAM, HR800) with 532 nm solid-state laser excitation.

### FDTD Simulation

Extinction spectra and electric field distributions were calculated using the three-dimensional FDTD method with the commercial Lumerical software package. The Au core and CNTS shell nanoparticles were covered by a perfectly matched layer boundary condition with a mesh size of 1 nm. A total field scattered field source was used with the normal incident over 400–1000 nm. The optical constant of gold (Au)^59^ and of CNTS was used from reported data.^60^

### Optoelectronic Measurements

The optoelectronic responses of the fabricated photodetectors were characterized by a probe station system (Keithley, 4200 SCS) using a light source of the visible region of wavelengths 405, 532, and 632 nm laser to excite the photoactive layer.

## Supporting Information Available

HAADF-STEM mapping of CNTS and Au/CNTS NCs, EDXS spectrum of CNTS and Au/CNTS NCs, XPS of CNTS NCs, Raman spectra of transfer graphene, scattering and absorption efficiency of CNTS and Au/CNTS NCs, device fabrication process, OM image of patterned graphene and the fabricated device, transient photocurrent response, EQE plots for the Au/CNTS/graphene photodetector at a 405 nm wavelength, device parameter calculation, and comparison table for different types of materials (PDF)
