# Supplementary material for: Superior Visible Photoelectric Response with Au/Cu2NiSnS4 Core–Shell Nanocrystals
Source: ACS Appl Mater Interfaces. 2024 Feb 26;16(9):12033–41. doi: 10.1021/acsami.3c17462 (PMC10921381; doi:10.1021/acsami.3c17462)
Supplement: Supplementary file 1 — am3c17462_si_001.pdf [file am3c17462_si_001.pdf]

Supporting Information

Superior Visible Photoelectric Response with Au/Cu_2NiSnS_4 Core-Shell Nanocrystals

Anima Ghosh[†,‡,Δ], Shyam Narayan Singh Yadav[§,Δ], Ming-Hsiu Tsai[‖], Abhishek Dubey[§], Chih-Ting Lin[‖], Shangjr Gwo[¶,#], and Ta-Jen Yen[*,§]*

[†]*Institute of Atomic and Molecular Sciences, Academia Sinica, Taipei 106, Taiwan R.O.C.*

[‡]Department of Physics, School of Sciences and Humanities, SR University, Warangal, India-506371.

[§]Department of Materials Science and Engineering, National Tsing Hua University, No. 101 Section 2, Kuang Fu Road, Hsinchu City 300, Taiwan R.O.C.

[‖]Graduate Institute of Electronics Engineering, National Taiwan University, Taipei 106, Taiwan, R.O.C.

[¶]Department of Physics, National Tsing Hua University, Hsinchu City 300, Taiwan R.O.C.

[#]Research Centre for Applied Science, Academia Sinica, Taipei 115, Taiwan R.O.C.

*Email: tjyen@mx.nthu.edu.tw

[Δ]A.G. and S.N.S. Yadav contributed equally to this paper.

HAADF-STEM Mapping of CNTS and Au/CNTS Nanocrystals

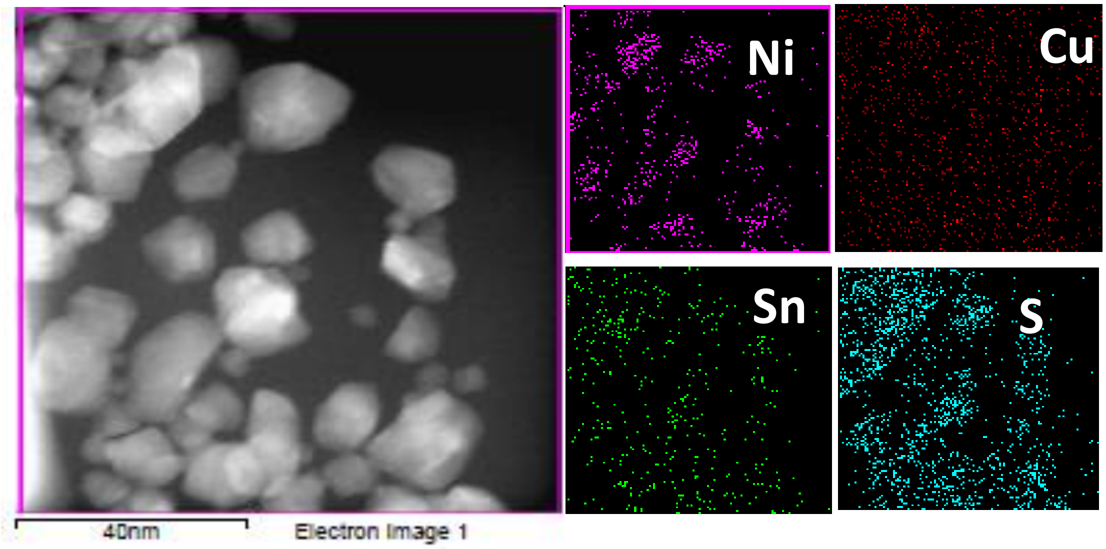

Figure S1: HAADF-STEM mapping of CNTS NC showing the presence of Ni, Cu, Sn and S.

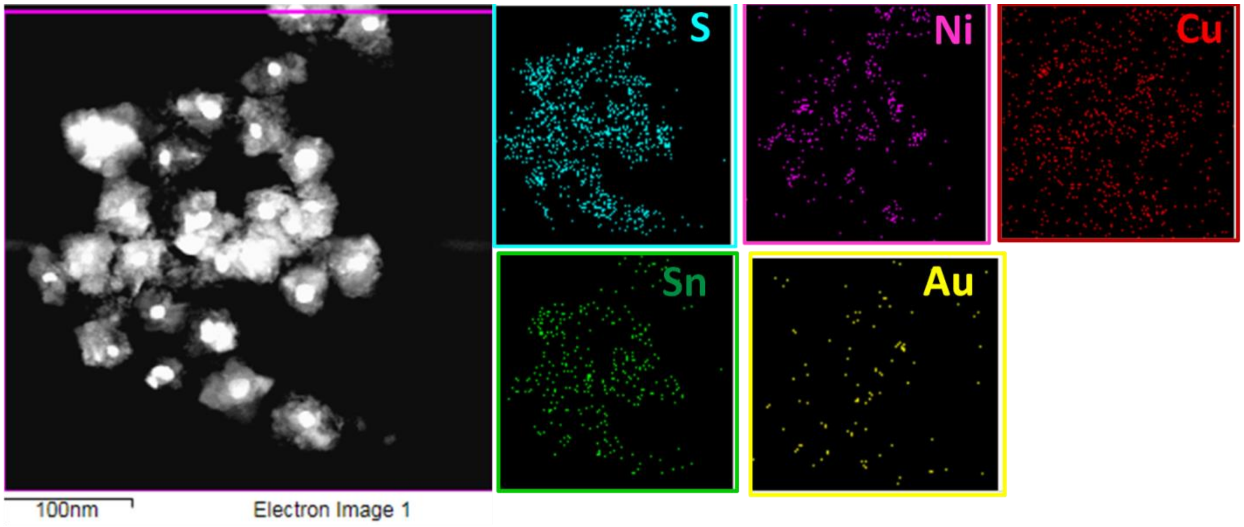

Figure S2: (a) HAADF-STEM mapping of Au/CNTS NC showing the presence of S, Ni, Cu, Sn and Au.

EDX of CNTS and Au/CNTS Nanocrystals

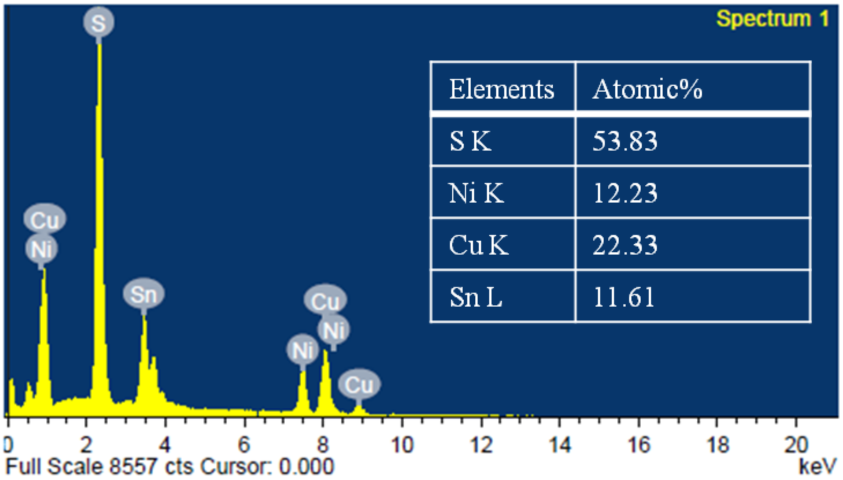

Figure S3: Energy dispersive X-ray (EDX) analysis of CNTS NCs.

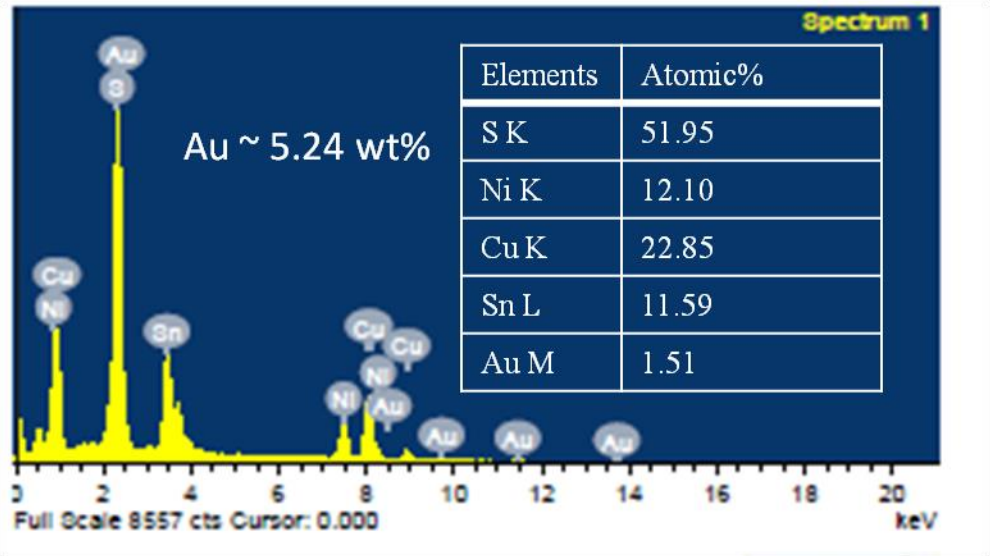

Figure S4: Energy dispersive X-ray (EDX) analysis of Au/CNTS NCs.

XPS of CNTS Nanocrystals

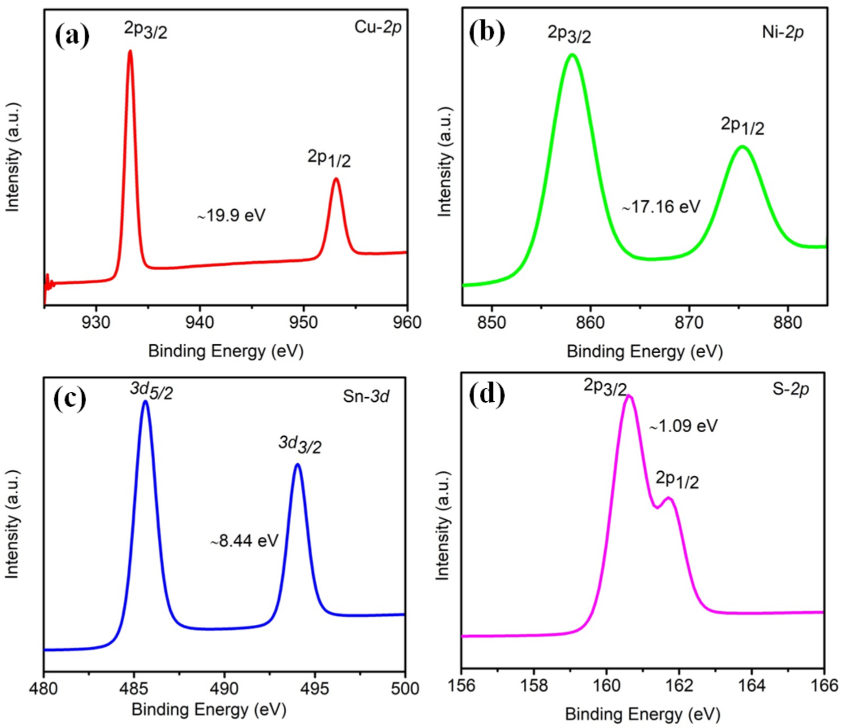

Figure S5: X-ray photoelectron spectroscopy spectra analyses of CNTS (a) Cu *2p*, (b) Zn *2p*, (c) Sn *3d, and* (d) S *2p levels*.

Raman Spectra of CVD-Grown Graphene

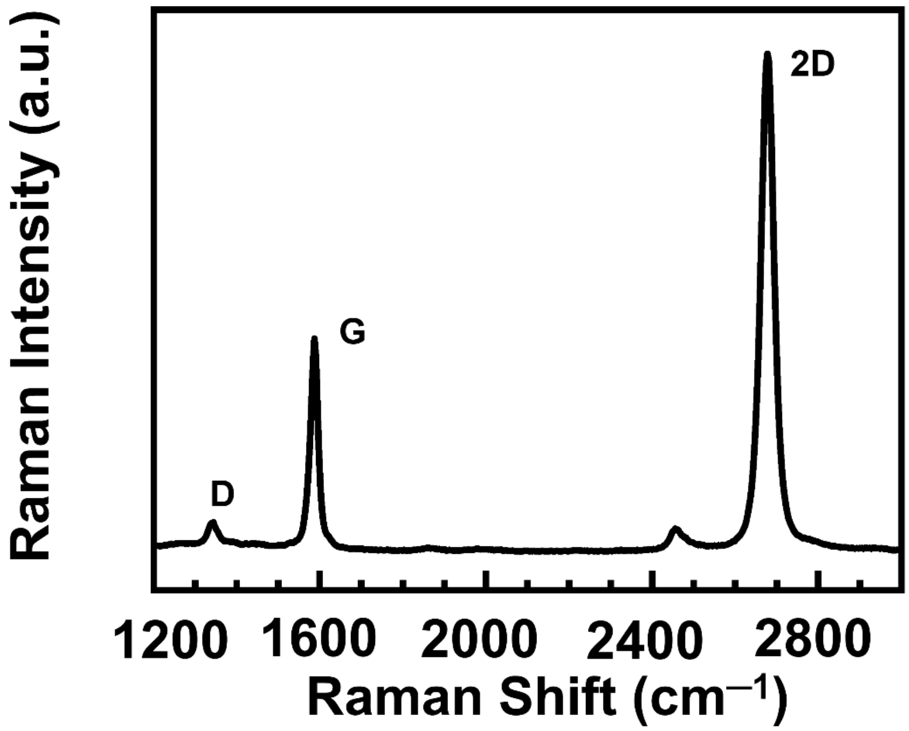

Figure S6: Raman Spectra of CVD-grown monolayer graphene. The peaks D, G, and 2D bands at 1340 cm^{-1}, 1580 cm^{-1}, and 2690 cm^{-1}, respectively. The D band represents bonds involving non-sp^2- hybrids C atom; however, G and 2D bands are characteristic of the C-C band of sp^2 hybridization of C atoms, consistent with the literature.[1]

Scattering and Absorption Efficiency of CNTS and Au/CNTS Nanocrystal

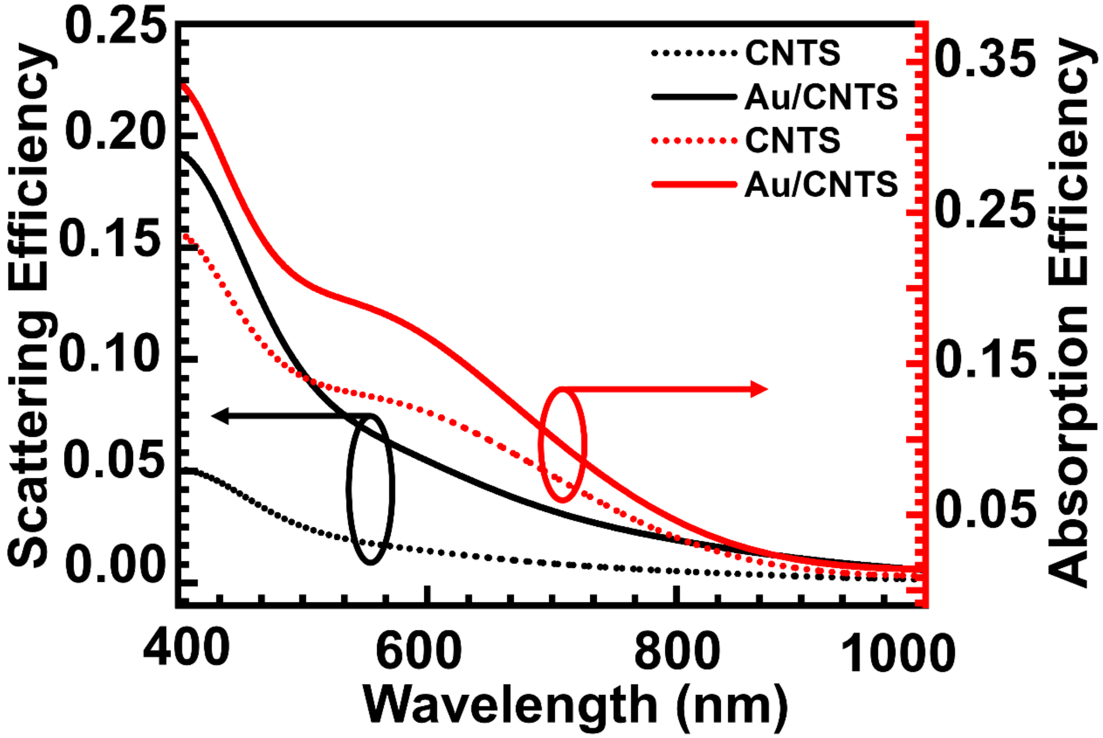

Figure S7: Finite difference time domain (FDTD) simulated scattering and absorption efficiency of CNTS and Au/CNTS NCs.

Device Fabrication Process

For device fabrications, at first, (Step I) the thermal grown SiO_2 285 nm on p+ doped silicon (Si) SiO_2/p^+-Si substrate was washed with acetone, and isopropyl alcohol for 5 minutes and rinsed with deionized water for 1 minute, followed by drying with N_2 and baking for 5 minutes on a hot plate at 110 °C for 5 min. (step II) Graphene was transferred by a similar method as discussed in the literature[1]. After that, the graphene channel was patterned with a 70×70 μm^2 Area size using UV photolithography followed by the O_2 plasma etching process shown in the device fabrication process in step III-VI at optimized 50-watt RF power with 100 sccm flow rate for 5 minutes.[2] The Drain and source electrodes were patterned using UV photolithography and deposited with Cr/Au (5/50nm) using the E-gun evaporation technique as shown in steps VII and VIII. To remove moisture, the fabricated substrate was preheated at 100°C for 5 min on a hot plate in the air. Pristine (e.g., CNTS) and Au/CNTS solution (disperse in n-hexene) was spin-coated at 1000 rpm for 30 secs separately and dried at 100 °C for 2 min (step IX). The spin coating process was repeated three times to get desired thickness of the photoactive layer at top of the graphene and labeled as CNTS and Au/CNTS.

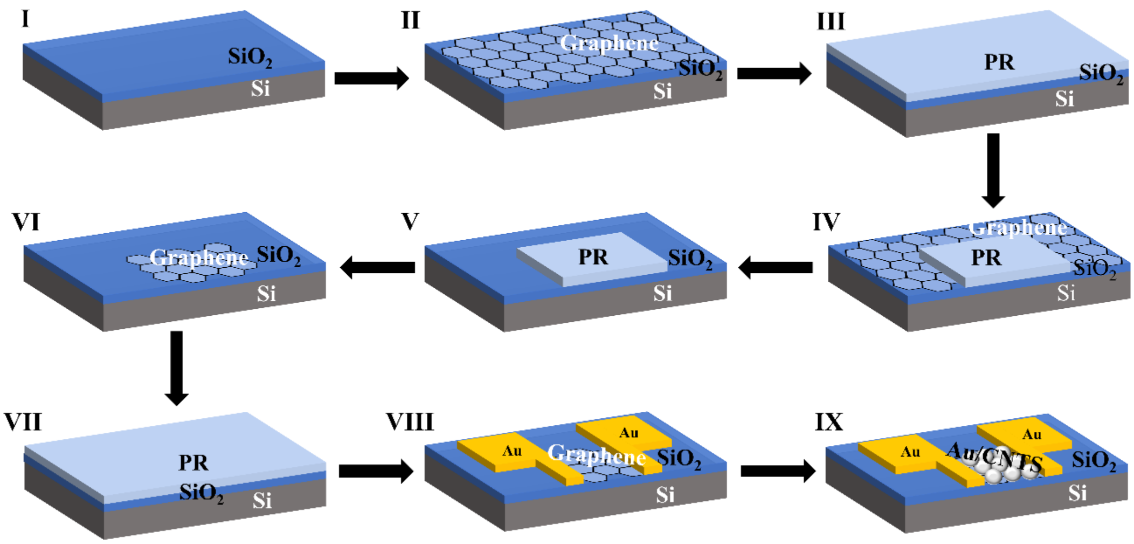

Figure S8: Device Fabrication Process Flow.

Optical Microscope Image of Fabricate Device

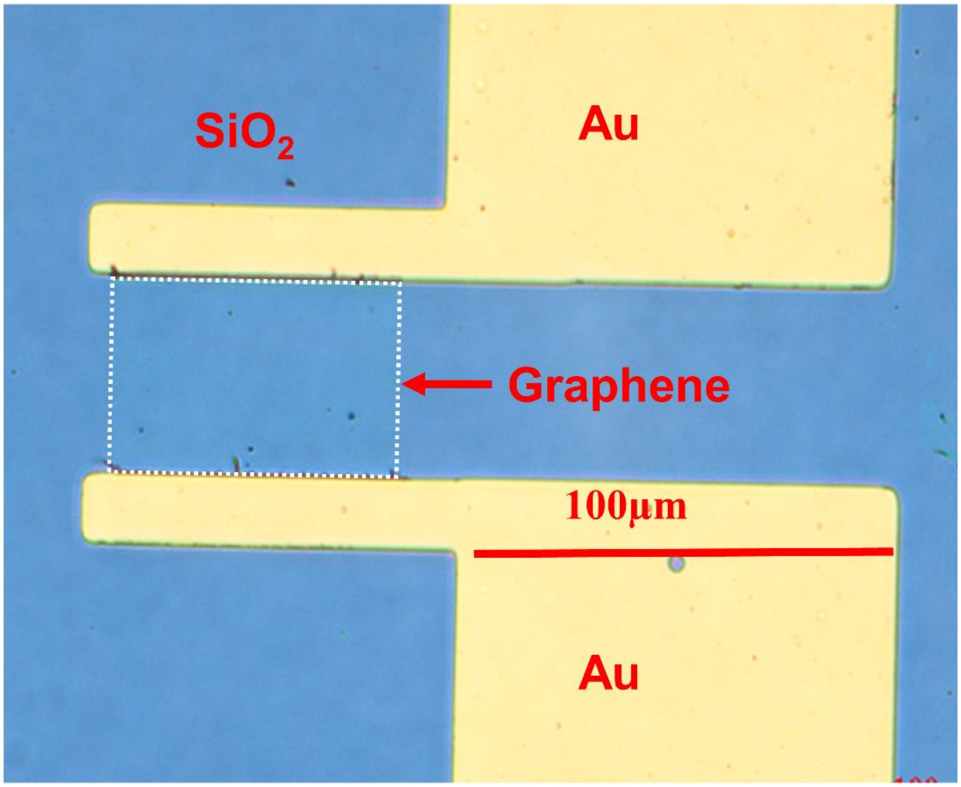

Figure S9: Optical Microscope image of Graphene/SiO_2/Si with deposited au electrode at 100 μm scale.

Transient Photocurrent Response of CNTS and Au/CNTS Photodetector

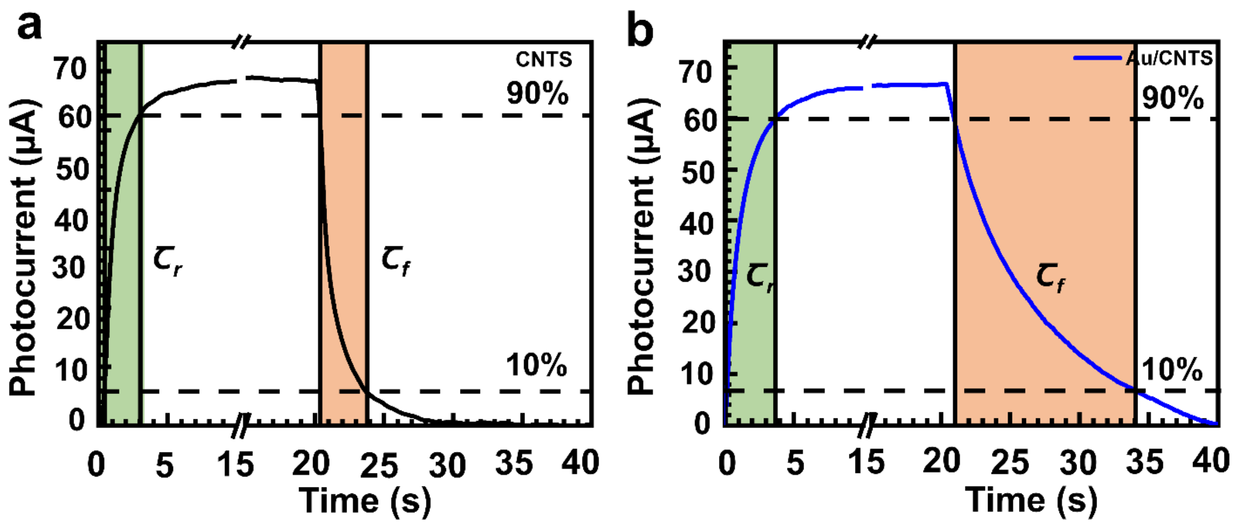

Figure S10: Transient photocurrent response for (a) CNTS, and (b) Au/CNTS at constant laser illumination power of 405 nm incident wavelength.

EQE of Au/CNTS Photodetector

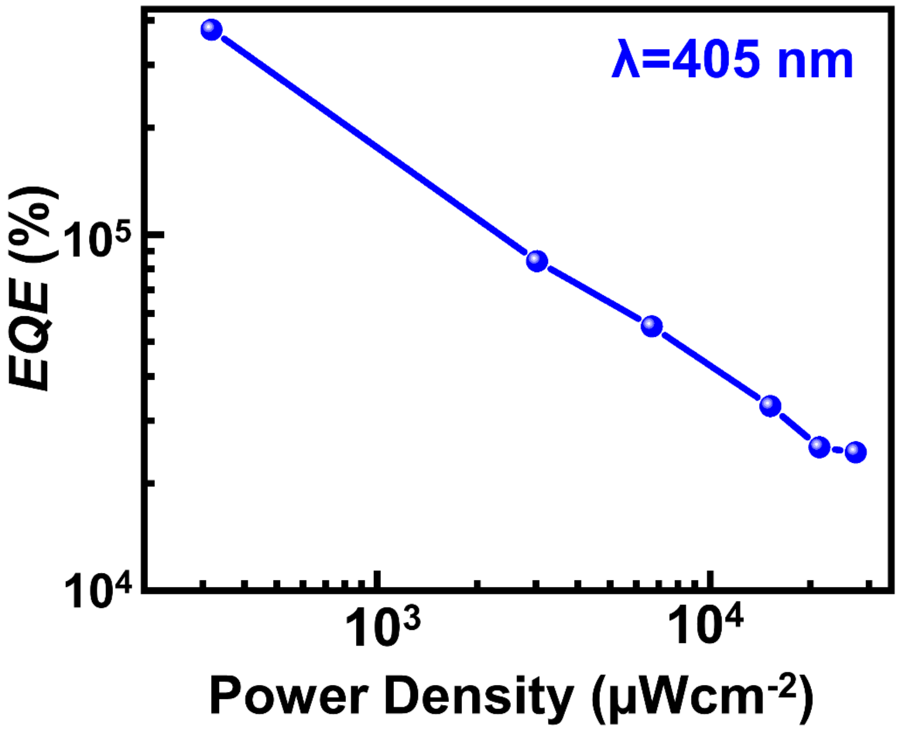

Figure S11: External quantum efficiency (EQE) with power density for Au/CNTS-based photodetector at incident wavelength of 405 nm.

Device Performance Calculations

The calculation of device parameters (Responsivity (R), Detectivity (D*), and EQE for Au/CNTS/Gr-based Photodetector

The parameters of the Au/CNTS/Gr-based Photodetector device was obtained under an illuminating wavelength of 405 nm with 10 μW power, and an applied bias voltage of 2 V.

The photoresponsivity (*R*) for NCs-based PD can be estimated using equation (1);

$$\boldsymbol{R} = \frac{I_{photo}}{P}, \tag{1}$$

where I_{photo} is the photocurrent ($I_{photo} = I_{light} - I_{dark}$), and, P is the illuminated power to the device [(laser power/laser spot area) × device active area].

R = (11.92987 μA ×0.0314 cm^{-2}) / (10 μW× 3048 ×10^{-8} cm^{-2}) = **1228.995 AW^{-1}**

The Detectivity (*D**) for Au/CNTS/Gr-based Photodetector can be calculated using equation (2);

$$\boldsymbol{D}^{*} \frac{R_{\lambda}}{\sqrt{\frac{2qI_{dark}}{A}}}, \tag{2}$$

where R_λ, q, I_{dark}, and A, denote the responsivity at a wavelength of λ, charge, dark current, and active area of the device, respectively. The active area of the device was measured to be 3048 μm^2.

D* = 1228.995 AW^{-1}× (3048 ×10^{-8} cm^2)$^{1/2}$/ (2× 1.6×10^{-19} ×0.000376182 A)$^{1/2}$ = **6.1842×10^{11} Jones.**

The specific detectivity (*D**) of the Au/CNTS-based photodetector can be also calculated using Equation 3;

$$D^* = \frac{\sqrt{AB}}{NEP}; \quad (3)$$

Where A is the active area, B is the bandwidth which is inversely proportional to response time, and NEP is noise equivalent power.

NEP can be expressed as Equation 5;

$$NEP = \frac{I_N}{R}; \quad (5)$$

Here, I_N represent the noise current, and R is the photoresponsivity of the photodetector. Further, I_N is expressed as $I^2{}_N = 2eI_D B$, where, e is the electric charge, I_D represent the dark current, and B denotes the bandwidth.

Now $B = 1/\tau$ = 1/3.4 sec. = **0.2941 S^{-1}**

$I_N = (2\times1.6\times10^{-19}\times3.76182\times10^{-4}\text{ A}\times0.2941\text{ S}^{-1})^{1/2}$ = **0.595×10^{-11} A**

Next, NEP can be calculated by $NEP = 5.95\times10^{-12}\text{A}/1.228\times10^{3}\text{ AW}^{-1}$ = **4.845×10^{-15} W**

Now, specific detectivity can be calculated as $D^* = (3048\times10^{-8}\text{ cm}^2\times0.2941\text{ Hz})^{1/2}/(4.845\times10^{-15}\text{ W})$ = **6.1796×10^{11} cm $Hz^{1/2}W^{-1}$ (Jones)**

The EQE for Au/CNTS/Gr-based Photodetector can be calculated using equation (4);

$$\boldsymbol{EQE} = \frac{(hcR_\lambda)}{q\lambda}, \quad (4)$$

where h, c, R_λ, q, and λ, indicate the Planck constant, speed of the light, responsivity at the wavelength of λ, charge, and wavelength of the incident light, respectively.

Here hc/q = 1240

EQE = $((1228.995\text{ AW}^{-1}\times1240\times100)/405)$ = **3.76285×10^{5} %**

Table S1. Photodetector performance comparison table for a different type of materials

Type of Photodetector	Response Range	Responsivity [AW^{-1}]	Detectivity [Jones]	EQE [%]	Bias [Volt]	References
CuO/Si Nanowire	405-1064	0.39 $x10^{-3}$ (405 nm, 0.048Wcm^{-2})	3.0 $x10^{9}$	30–35	0	[3]
ZnO/WS_2	400-660	1.75 (660 nm, 0.5 Wcm^{-2}	–	–	1	[4]
$ZnO-Co_3O_4$ core shell nanowire	400-700	$21.8x10^{-3}$	$4.12x10^{12}$	–	0.1	[5]
PbS $QDs/CdS_{0.33}Se_{0.67}$ nanosheet	405-1065	$1.45x10^{3}$ (405 nm, 0.01 $mWcm^{-2}$)	–	$1.05x10^{15}$	5	[6]
$Cds-CdS_xTe_{1-x}$-CdTe core-shell nanobelt	355-785	$1.5x10^{3}$ (405 nm, $2.78x10^{-7}$ Wcm^{-2})	–	–	5	[7]
CZTS (Na doped)	532	51	–	–	-	[8]
CZTS	532	14.6mA	3.5×10^{9}	3.5%		[9]
FDS02, Si-based photodiode	400-1100	0.146 (400 nm)	–	–	5	Thorlabs
FDS10×10, Si-based photodiode	340-1100	0.2 (400nm)	–	–	5	Thorlabs
CNTS/Graphene	405-632	0.6×10^{3} 405 nm	3.36854×10^{11}	2.0×10^{5}	2	This work
Au/CNTS core-shell/Graphene	405-632	1.2×10^{3} 405 nm	6.18×10^{11}	3.7×10^{5}	2	This Work

REFERENCES

(1) Lin, C.-H.; Tsai, M.-S.; Chen, W.-T.; Hong, Y.-Z.; Chien, P.-Y.; Huang, C.-H.; Woon, W.-Y.; Lin, C.-T. A low-damage plasma surface modification method of stacked graphene bilayers for configurable wettability and electrical properties. *Nanotechnology* **2019**, *30* (24), 245709.

(2) Samukawa, S.; Hori, M.; Rauf, S.; Tachibana, K.; Bruggeman, P.; Kroesen, G.; Whitehead, J. C.; Murphy, A. B.; Gutsol, A. F.; Starikovskaia, S.; et al. The 2012 Plasma Roadmap. *J. Phys. D: Appl. Phys.* **2012**, *45* (25), 253001.

(3) Hong, Q.; Cao, Y.; Xu, J.; Lu, H.; He, J.; Sun, J.-L. Self-Powered Ultrafast Broadband Photodetector Based on p–n Heterojunctions of CuO/Si Nanowire Array. *ACS Appl. Mater. Interfaces.* **2014**, *6* (23), 20887-20894.

(4) Butanovs, E.; Vlassov, S.; Kuzmin, A.; Piskunov, S.; Butikova, J.; Polyakov, B. Fast-Response Single-Nanowire Photodetector Based on ZnO/WS2 Core/Shell Heterostructures. *ACS Appl. Mater. Interfaces.* **2018**, *10* (16), 13869-13876.

(5) Ghamgosar, P.; Rigoni, F.; Kohan, M. G.; You, S.; Morales, E. A.; Mazzaro, R.; Morandi, V.; Almqvist, N.; Concina, I.; Vomiero, A. Self-Powered Photodetectors Based on Core–Shell ZnO–Co3O4 Nanowire Heterojunctions. *ACS Appl. Mater. Interfaces.* **2019**, *11* (26), 23454-23462.

(6) Peng, M.; Xie, X.; Zheng, H.; Wang, Y.; Zhuo, Q.; Yuan, G.; Ma, W.; Shao, M.; Wen, Z.; Sun, X. PbS Quantum Dots/2D Nonlayered CdSxSe1−x Nanosheet Hybrid Nanostructure for High-Performance Broadband Photodetectors. *ACS Appl. Mater. Interfaces.* **2018**, *10* (50), 43887-43895.

(7) Tang, M.; Xu, P.; Wen, Z.; Chen, X.; Pang, C.; Xu, X.; Meng, C.; Liu, X.; Tian, H.; Raghavan, N.; et al. Fast response CdS-CdSxTe1−x-CdTe core-shell nanobelt photodetector. *Science Bulletin* **2018**, *63* (17), 1118-1124.

(8) Singh, O. P.; Sharma, A.; Gour, K. S.; Husale, S.; Singh, V. N. Fast switching response of Na-doped CZTS photodetector from visible to NIR range. *Solar Energy Materials and Solar Cells* **2016**, *157*, 28-34.

(9) Gour, K. S.; Singh, O. P.; Bhattacharyya, B.; Parmar, R.; Husale, S.; Senguttuvan, T. D.; Singh, V. N. Enhanced photoresponse of $Cu_2ZnSn(S, Se)_4$ based photodetector in visible range. *J. Alloys Compd.* **2017**, *694*, 119-123.
